# Lessons learned from concerned significant others: a qualitative analysis on involvement in services for young adult opioid use disorder

**DOI:** 10.3389/fpubh.2025.1512529

**Published:** 2025-07-17

**Authors:** Nicole P. Porter, Sean Dunnsue, Cori Hammond, Molly Bobek, Alexandra MacLean, Mari Watkins, Craig E. Henderson, Aaron Hogue

**Affiliations:** ^1^Family and Adolescent Clinical Technology & Science, Partnership to End Addiction, New York, NY, United States; ^2^Department of Psychology, Sam Houston State University, Huntsville, TX, United States

**Keywords:** young adult, concerned significant other, family, opioid use disorder, family involvement

## Abstract

**Introduction:**

Research, clinical wisdom, and government policy recommend family involvement in services for young adult (YA) opioid use disorder (OUD) to improve treatment outcomes. Moreover, research suggests YAs believe that family involvement is essential to OUD treatment and prefer greater involvement of their concerned significant others (CSOs), such as family members, romantic partners, and family-of-choice members in their care. Yet, CSOs are not routinely involved in OUD services for YAs. The main aim of this qualitative study is to learn from CSOs and YAs directly about their thoughts, beliefs, attitudes, and experiences with family-involved services.

**Method:**

We used convenience sampling to recruit 10 YAs (ages 24–36 years) who were in treatment for OUD and their CSOs (5 mothers, 2 grandmothers, 2 partners, 1 aunt) from two urban treatment centers. Using semi-structured interview guides, we conducted qualitative interviews with YAs and their CSOs to explore their experiences, feelings, and attitudes toward family involvement in services. Thematic content analysis started with deductive-dominant group consensus coding followed by matrix analysis to analyze themes in the context of CSO-YA dyads.

**Results:**

We identified five main themes: (1) CSO-YA relationships were resilient and motivated treatment and recovery, (2) CSOs believed in the importance of family involvement in services and experienced personal benefits by participating, (3) CSO involvement occurred on a continuum from facilitating treatment entry to systemic family therapy, (4) YAs identified CSOs who were supportive and encouraging of treatment even in the face of CSO barriers and challenges, and (5) YAs held accurate perceptions of their CSOs' MOUD attitudes and beliefs.

**Discussion:**

In this qualitative study we learned from YAs and CSOs themselves about the individual and relational benefits of family integration in services and replicated findings from previous research highlighting preferences for greater family involvement in OUD services. Clinical implications and recommendations for challenging barriers to relationship-oriented services and recovery planning for OUD are discussed.

## Introduction

Rates of opioid use among adolescents and young adults (YAs) remain high despite the known risks associated with opioid use and national efforts to increase prevention and treatment. Among YAs, rates of past year opioid use have been found to be as high as 32%, with rates of subsequent opioid misuse around 8% (1). Moreover, lethal overdoses related to opioids among this population have increased 268% from 1999 to 2016 (2), and up to two-thirds of overdose deaths among YAs involve opioids (3).

YAs are notoriously difficult to engage in treatment for opioid use disorder (OUD), and they tend to have worse outcomes compared to mature adults (4). Recent studies have shown that fewer than one-third of YAs currently receive adequate treatment (5, 6). Furthermore, although medication for OUD (MOUD) is the standard of care for youth OUD (7, 8), MOUD remains vastly underutilized in this age group (9, 10). For youth who do access MOUD treatment, most struggle with adherence, retention, and premature treatment termination (9, 11, 12).

Involvement of concerned significant others (CSOs) is a key indicator of high-quality substance use services of all kinds including OUD and MOUD (13, 14). In practice, CSOs of YAs often tend to be caregivers [e.g., (15, 16)]; however, they may also be other loved ones and family-of-choice members like friends and partners. CSOs can be involved in substance use services across the treatment services continuum, from learning about services and facilitating treatment entry, to attending family groups and sessions to learn psychoeducation and new family-oriented behavioral skills (14, 17). Moreover, CSOs can be engaged in services to meet their own needs including self-care and coping with the emotional burden of caring for loved ones struggling with addiction (16, 18). Research indicates CSO involvement in substance use services is associated with greater treatment engagement (16), reduced rates of drop out (41), increased length of sobriety (19), increased MOUD adherence (20, 21), and decreased number of relapses (22). Furthermore, new research suggests YAs may actually prefer greater involvement of their CSOs in their treatment (23). Unfortunately, family and CSO involvement in OUD services is inconsistent (24, 25), a significant problem that research is only beginning to address.

### Barriers to CSO involvement in services

Barriers to family and CSO involvement in services exist across ecological systems. A 2024 scoping review by Tham and Solomon (26) of barriers to family involvement in behavioral services for severe mental illness identified barriers embedded in organizational culture and structure, societal attitudes, provider training and education, and relationship dynamics among patients, their families, and providers. Little is known about the barriers to family and CSO involvement in services for YA OUD specifically, although the scant research to date suggests barriers may be similar.

Limited training opportunities for providers to learn techniques to collaborate with family members, along with lack of administrative priorities for providing family services, have plagued behavioral health services (26) and may also impact family involvement in YA OUD services. In one study, YA OUD providers reported inadequate training, education, and experience engaging and collaborating with families, as well as concerns about confidentiality (25). These barriers coincide with programmatic limitations including scarce opportunities for family members to participate in services, lack of provider time for family outreach, limited staff for family groups, and restrictions on holding counseling sessions that involve family members, as well as administrative barriers related to appointment scheduling (25).

Societal attitudes and stigma are other known barriers to family members seeking services and participating in services for their loved ones (27, 28). This is especially true for family members of individuals struggling with OUD. Family members are reluctant to pursue supportive services due to experiences of stigma and shame (29). Concurrently, stigmatized clients and patients are reluctant to invite their family members to participate in services for the same reasons (30).

Even more concerning is evidence that providers may hold negative attitudes and perceptions about family members and the efficacy of family involvement in services, curbing the extent to which they pursue opportunities to collaborate with CSOs. In one study exploring attitudes among YA OUD providers, providers reported seeing less value in collaborating with families for patients over the age of 18 related to assumptions about lower reliance on the family system among this age group (31). Another study assessing experiences of family members found that not only were family members dissatisfied with their involvement in services, but also they felt dismissed and patronized by providers (29).

Finally, poor access to care is a longstanding problem for behavioral health treatment that persists for YA OUD services. Family members experience challenges navigating the treatment system, in particular understanding the available services to support their loved one (29). The emotional toll of caring for family members struggling with OUD (32) and lack of knowledge about services available to support themselves (29) further impairs access to care for CSOs.

## Current study

The extant research on family and CSO involvement in OUD services for YAs focuses on experiences reported by YA OUD providers and YA patients. There are only a handful of studies seeking the perspectives of CSOs themselves (e.g., 29). The main aim of this qualitative study is to learn from CSOs and YAs directly about their thoughts, beliefs, attitudes, and experiences with family-involved services. This qualitative study has two main innovations. First, it is among the first to interview CSOs about navigating OUD services and their opinions about the role of CSOs in YA OUD services and recovery processes. Learning from stakeholders who are the target of services—in this case, family-focused interventions—is directly relevant to developing training and implementation strategies to improve intervention uptake. Second, this study is located in supporting relationship-oriented approaches and highlights the value of the social context in treatment and recovery planning for YAs. Supportive family relationships and social support are key forms of recovery capital for YAs that help to mitigate their unique developmental vulnerabilities and risk factors (6).

## Method

### Study design and population

We conducted qualitative interviews with YAs and their CSOs. YAs were recruited from two OUD treatment centers housed under one drug and alcohol treatment program in a large metropolitan area. Both treatment clinics offered opportunities for CSO participation in family education and support groups, check-ins with the treatment team, and family sessions when patients expressed desire to have family members involved and provided consent for counselors to contact them. All study protocols were approved by the governing IRB.

### Eligibility and recruitment

We used convenience sampling to recruit 25 YAs over a 13-month period (November 2022 to December 2023). At each treatment center, an in-house research assistant identified and referred to the study team patients between the ages of 18 and 30 who were currently or previously enrolled in the center's MOUD treatment program. Patients were required to be able to speak and understand English and provide informed consent. YAs who consented to participate completed a brief demographic and opioid use history survey via Qualtrics and an in-person qualitative interview during a subsequent site visit by the research team. At the end of each interview, YAs were asked for permission to contact their CSO whose role in treatment had been discussed as part of the interview. CSOs were contacted by and interviewed over a 14-month period (December 2022 to January 2024). Twenty of the 25 YA participants gave permission to contact their CSO for a follow-up interview and provided contact information. We were unable to reach 12 CSOs using the contact information provided by the YA. Because the YA's participation in the study had ended, we did not recontact YAs for new or additional contact information for their CSO. Thirteen CSOs (65%) were reached by telephone and expressed interest in participating in the study, however 3 CSOs were subsequently unable to be reached for consent and enrollment. The 10 remaining CSOs consented to participate, completed a brief demographic and substance use and treatment history questionnaire via Qualtrics, and were interviewed via video conference. All YA and CSO interviews were conducted by the same two trained research assistants (authors SD and MW). Interviews lasted ~45 min and YAs and CSOs each received a $40 Amazon gift card voucher as compensation for interview completion.

### Data collection and analysis

Semi-structured interview guides were used for qualitative interviews for both YAs and CSOs (see Appendix A for CSO sample). The interview guides consisted of open-ended questions and prompts designed to lead the interviewer and participant through different topics and content areas while allowing each participant space to tell their unique story. Questions explored domains relevant to OUD services and common barriers to family involvement in behavioral services: (1) past and current treatment experiences, (2) perspectives on treatment goals including MOUD treatment, (3) attitudes and beliefs about family involvement in services and recovery planning for YA OUD, (4) family relationships and dynamics including the relationship between CSOs and YAs, and (5) barriers to family participation in services. All interviews were audio recorded and transcribed verbatim by the study team. All identifiers were removed prior to coding. For this study we used a random name generator of the most common names in the United States to create gendered pseudonyms for participants to conceal identifiers (e.g., race/ethnicity).

We used mixed deductive-inductive qualitative content analysis procedures (33) with team-based codebook refinement (34). The coder cohort consisted of 6 co-authors. Four identified as White, and two as Multirace. Four identified as cisgender female, one as cisgender male, and one as non-binary. Three had master's degrees and one had a doctoral degree. The lead qualitative interviewer (author SD) created deductive codebooks based on the questions asked and responses commonly collected during interviews. The study team reviewed the proposed codebooks and engaged in an iterative process to refine codes and definitions for clarity and precision before beginning to code.

Coding started with group consensus coding, whereby each member of the coding team independently coded a study transcript and participated in a live coding group to discuss codes and reach agreement. Next, we double-coded study transcripts and continued live coding groups to reach agreement. During live meetings, coders completed a detailed segment-by-segment review of the entire transcript as well as the application of codes in each segment. We continued iterative refinement of existing codes and definitions while also using inductive qualitative content analysis procedures to add new codes and definitions to the codebooks to reflect unanticipated emerging themes. After intercoder reliability was established, coding continued independently with weekly coding meetings to maintain reliability. All coding was conducted using ATLAS.ti software version 24 (35). Matrix analysis was used as a complementary strategy to synthesize and interpret coding results using a grid that listed interview participants along the x-axis and concepts along the y-axis to show which concepts emerged in each interview as well as the frequency of each concept across the study sample (36). To analyze CSO data in the context of YA data, each column represented a CSO-YA dyad rather than an individual participant. With these analytic procedures, we learned about CSO past and current experiences accessing and participating in services for their loved ones in the context of YA experiences and opinions on related topics.

### Techniques to enhance coding trustworthiness and reflexivity

Coding reliability was focused on internal reliability and the quality, authenticity, and truthfulness of our findings (42). Therefore, we favored techniques to enhance coding trustworthiness rather than quantitative measures of inter-coder agreement, consistent with current recommendations (37). The coding team mitigated potential coding bias during thematic analysis to enhance trustworthiness and credibility in four main ways. First, coders received training on qualitative data analysis. Second, as described above, 20% of transcripts were coded by two or more coders to enhance reliability. Third, two members of the authorship team who were not involved in coding and thematic analysis independently reviewed all themes and supporting quotations. Finally, we followed the Standards for Reporting Qualitative Research [SRQR; (38)].

The coding and authorship teams engaged in team-reflexive discussions to understand each person's position within the research, experiences and beliefs about the content matter, experiences and orientation toward qualitative research, and favored data analysis procedures throughout the design, execution, and reporting of this qualitative study (39).

## Results

### Participants

Participants were 10 YA-CSO dyads. YAs were on average 27 years old (SD = 4.1, range = 20–36; 70% age 20 to 30). Self-identified race was 50% Black or African American (*n* = 5), 30% White (*n* = 3), 20% Multirace or another race (*n* = 2). Twenty percent (*n* = 2) identified as Latino/a/x, Spanish, or Hispanic. Participants were predominantly male (60%; *n* = 6). CSOs were five mothers, two grandmothers, two partners, and one aunt. Self-identified race and ethnicity was 50% White (*n* = 5), 30% Black or African American (*n* = 3), and 20% Latino/a/x, Spanish, or Hispanic. Participants were predominantly female (80%; *n* = 8). Three CSOs (30%) reported a history of substance use disorder and of those 2 reported receiving substance use disorder treatment. Demographic data were missing for 2 CSOs.

We identified five main themes related to study aims. Each theme is described below along with exemplar statements drawn from interviews and organized in rows by dyad.

### Theme 1: CSO-YA relationships are resilient and a source of strength and motivation for treatment and recovery

Among the 10 CSO-YA dyads, 8 dyads shared stories centered on the resiliency of their relationship, describing their connection as one that has withstood conflict and challenges associated with substance use and relapse (e.g., stealing; Seth and Barbara, fighting; Megan and Amy). Many YAs and CSOs described feeling more connected with one another having navigated and overcome difficult experiences together (Table 1).

**Table 1:** Exemplars for Theme 1: CSO-YA relationships are resilient.

**YA**	**CSO**
*So, our relationship at first was like rocky…we would go back and forth, I would yell, we'll be arguing, but it, you know, as time went on and I started to like appreciate things a lot more or appreciate that she's not here to like, you know, put me down, she's just here to help or she just cares*. - Seth (age 25-30)	*YA started taking stuff. Stealing money and taking stuff from my daughter and stuff like that. That kind of changed our relationship because he couldn't be trusted…He needs someone in his corner. When the therapist wants me to meet with him and her, I try to make myself available for that treatment. I call to see how he's doing. I want him to feel like he's loved*. - Barbara, Seth's grandmother
*[Our relationship is] good. Well, it's actually great. It's better now because before was like when I was dealing with the drugs. I had like a lot of moments that weren't good, so I kind of took my anger out on her with the withdrawals and when I wanted to use and stuff like that, so..*. - Megan (age 25-30)	*Just as a mom being concerned, I would ask her questions and she would come out and tell me. [YA] would always be truthful to you and she‘ll tell you the truth so we have a great relationship. Of course, sometimes we might get a little rocky it's because maybe she's using or whatever, but after that she'll come back to me and say mom I'm sorry I didn't mean to say it in that way, now I understand what you were saying*- Amy, Megan's mother
*When you have family involved in treatment, especially if you‘re doing like a group therapy thing like I was, it helps – it helped me kind of want to get clean 'cause I didn't before, but through doing like those family things that kind of – I realized more how it was impacting my relationships, especially with my family which made me want to stop, whereas I didn't before*. - Chris (age 25-30)	*Well you know, interestingly, although I wouldn't recommend going through a substance use issue with a family member as an ideal form of bonding, I feel like overall it has had that kind of effect in terms of I think that the process of going through treatment, and most especially intensive counseling…I think that a biproduct of this experience is that we are able to be more open and honest with one and other, and he's able to share his thoughts and feelings about things, you know, and especially about substance use*. - Alana, Chris's mother

Eight YAs described ways in which family relationships, and particularly the relationship with their CSOs, was a source of motivation for pursing abstinence and recovery goals. These YAs all had CSOs who held compassion for how difficult addiction can be and believed that supporting their loved one was crucial to their treatment and recovery (Table 2).

**Table 2:** Exemplars for Theme 1: CSO-YA relationships are a source of motivation for treatment.

**YA**	**CSO**
*Yeah, I think I think like, the biggest thing, like that helped me get sober I think was the relationship with my family because I really wanted to get sober and I really wanted to, but I, the cravings for fentanyl are so strong, that like, it kind of just overpowers any thinking that you really have*. - Grayson (age 25-30)	*I feel like with no family support, nobody on your side, being painted this horrible person, when you're in addiction, is probably one of the worst things you can do. Like it's just kicking somebody while they're down already. You need to lift people up, not shit on them while they're already in a hole, you know what I mean? I had some bad experiences, I'm just happy that I can give him the supports he deserves, because I don't feel like anybody deserves that type of treatment*. - Lucy, Grayson's mother
*I'm really excited because, you know, she's never agreed to come talk to my counselor for my treatment before. She just said, “I know what you need to do, you know what you need to do. It's just the kind of doing it.” But this time she's willing to come talk to my counselor and all that, so*. - Kelly (age 25-30)	*You can love her, and there's a way to love someone with an addiction problem, but they don't want to do it that way. So, I'm not gonna give up on [YA], you know. There's, you can love them, and you can give them tough love, but you don't have to give up on her either at the same time*. - Janis, Kelly's aunt

### Theme 2: CSOs believed in the importance of family involvement in services and experienced personal benefits from participating in services for their loved one

Every CSO believed family involvement in services for YAs is essential to OUD treatment. When asked, 8 out of 10 CSOs endorsed that families have a responsibility to help and support their loved one in treatment and recovery. Four CSOs elaborated on what it might be like for someone in treatment without family support, speaking to potential YA isolation and hopelessness (Table 3).

**Table 3:** Exemplars for Theme 2: CSOs believed in the importance of family involvement in services.

**CSO**
**Family involvement in services**
*I feel like when someone's in your family, when they‘re in your inner circle, it's your job to support their individual goals, and it's also their job to support the best interest of the family. So, there's that interconnectedness. So, yeah, I feel like, you know, it's definitely - there's definitely work involved if you're a family member supporting someone in recovery. There is work involved, there's time involved, there's heartache involved, and disappointment involved - but there's also joy, and successes to be celebrated, and positivity*- Alana, Chris's mother
*I think family is responsible for helping them. If you love them, that person...any person that is using and they have family that loves them, that's an automatic. Family is responsible. If that person is going to be in your life, it's your duty to try and help them*. - Karen, Ann's mother
*Well I don't think that's true at all, I think when you're addicted it affects the family. It's not only their problem, it's everybody's problem. Just like I had a sister who had cancer, it wasn't just her problem it was the family's problem. We all helped. So that's no different*. - Kristen, Martin's mother
**No family involvement in services**
*I feel like with no family support, nobody on your side, being painted this horrible person, when you're in addiction, is probably one of the worst things you can do. Like it's just kicking somebody while they're down already. You need to lift people up, not shit on them while they're already in a hole, you know what I mean? I had some bad experiences, I'm just happy that I can give him the supports he deserves, because I don't feel like anybody deserves that type of treatment?*- Lucy, Grayson's mother
*I mean like, and we wonder why all these people are out here doing this stuff. Because some people don't have no family or nobody they can just come and say, you encourage me, you call and check up on me, you don't know how much that means to a person*. - Amy, Megan's mother
*I think it is kind of hard when you don't have family support because you figure why am I bothering? Who cares? If nobody cares, they won't care*. - Christine, Helen's mother

Every CSO described personal and relational benefits from participating in their loved one's services and supporting their recovery journey. CSOs shared about experiencing reassurance and relief from receiving updates on treatment progress by participating in family sessions and check-ins with the treatment team (*n* = 3; e.g., Barbara). Others reflected on achieving more open family communication (*n* = 3; e.g., Alana and Caitlin) and about personal growth like better understanding addiction, shame, and stigma (*n* = 3; e.g., Alana). Two CSOs disclosed their own lived experience with addiction and described the mutual benefits to their loved one's recovery and their own (e.g., Janis) (Table 4).

**Table 4:** Exemplars for Theme 2: CSOs benefited from participating in services.

**CSO**
*And then at some point through the process, and of course being involved in family therapy, there was one point in a session when [YA] said to me a year ago I couldn't have told you this or I couldn't have said this to you. And I don't even remember what the precise topic was, but then I suddenly became aware of my own growth through this process. So, it wasn't just [YA] who was benefiting from all of this, I was as well. And you know, he made it very plain to me that, you know, that I had become much more open to being able to hear the truth from him, and that I had, you know, become better able to accept it. To accept his truth and accept his reality. And you know, that - in that way I had also grown and it was really like an epiphany for me 'cause I hadn't really been realizing that*. - Alana, Chriss mother
*like being more involved because then I know what is going on. The therapist tells me some of the things going on and then he can tell me exactly what he's doing. I need to know. I need to make sure that he's staying on the right track*- Barbara, Seth's mother
*I like this new behavior of him right now. And I told him I just hope he keeps it up. And I hope he, whenever he needs help or, you know, I'm here. Like I told him I want us to communicate more and talk more. Like if somethings on your mind or something you have in mind or whatever it may be, I just want us to talk more and let him know I am here to help him. I'm not his enemy*. - Caitlin, Owen's partner
*For me I guess it also helps keep me reminded of staying clean for myself. I mean I don't want to end up that way either, you know. I've seen too many people relapse in my life. I don't want to ever relapse in my treatment plans. It helps, you know, it helps me feel good that I can help [YA] and her daughter*. - Janis, Kelly's aunt

### Theme 3: CSO involvement can occur on a continuum from facilitating treatment entry to systemic family therapy

CSO involvement in services and a family approach in general can be conceptualized as a continuum of family involvement interventions with an array of complementary treatment goals (13). CSOs were involved in supporting YAs entering treatment (*n* = 8) and also further along the continuum (*n* = 9), participating in individual YA treatment sessions and also family therapy sessions. Facilitating treatment included expressing the importance, urgency, and necessity of attending treatment as well as specific facilitative actions such as bringing the YA to treatment and allowing natural consequences (e.g., withholding money from a YA not attending treatment) (Table 5).

**Table 5:** Exemplars for Theme 3: CSOs facilitate treatment entry.

**CSO**
**Facilitating treatment entry**
*Yeah, I really insisted on it, you know. I just said that, you know, if you're going to continue to live in this home, that you know treatment isn't optional; it's something that you have to do, and... yeah. So, he, you know, was willing to embrace that and – which was really helpful. I know a lot of people – it's much harder to get their loved one into treatment*. - Alana, Chris's mother
*You need to go to rehab, I‘ll take you to rehab. You need to go to a meeting, I'll take you to a meeting. I'm not giving you no money. And then he finally knew the only person he had to call was me, to take him to rehab, because he had exhausted all other resources*. - Jackie, Lewis's partner
*I just said you're gonna have to go to rehab. You're gonna have to do something. It's either them drugs or your family, so you need to pick one because this is not gonna work..... So, and he actually called. He called [facility name]. The same day I told him you're gonna have to do something*. - Caitlin, Owen's partner

In addition to facilitating treatment, some CSOs participated with YAs in treatment. While only one dyad participated in regular (weekly) family therapy sessions in which relationship dynamics were addressed in addition to individual treatment goals, nine family members participated in the YA's treatment in meaningful ways. Importantly, YAs valued the ways that CSO participation included communication with their therapists or other treatment providers, as this open and bidirectional communication provided accountability, positive reinforcement for treatment adherence, and advocacy when encountering roadblocks. Just as CSOs played important and unique roles in facilitating YA treatment entry, ongoing CSO participation supported ongoing YA treatment attendance and treatment outcomes (Table 6).

**Table 6:** Exemplars for Theme 3: CSO involvement can occur on a continuum.

**YA**	**CSO**
**Participating along continuum**	
*She's in like all the sessions like the every - we have a family session, once a month. And she's like, also, even still, now even though I have a new therapist now, but she's in the group message. Like, whenever we talk with me and my therapist, and my mom is in the chat as well. And I guess they let her know about the drug tests too that I get once month*. - Grayson (age 25-30)	
*They asked me if I wanted anybody to be a part of my treatment plan and I said my grandmother ‘cause I know, you know, she wants to be there to like talk to me whenever 'cause she knows I get irritated with like certain things that people will say or how people approach certain situations. And she's just there to help out and, you know, also just to learn, you know, see how I'm doing, you know what I mean*- Seth (age 25-30)	*I like being more involved because then I know what is going on. The therapist tells me some of the things going on and then he can tell me exactly what he's doing. I need to know*. - Barbara, Seth's grandmother
*When you have family involved in treatment, especially if you're doing like a group therapy thing like I was, it helps... I realized more how [SU] was impacting my relationships, especially with my family, which made me want to stop, whereas I didn't before*. - Chris (age 25-30)	*It's been pretty intensive because he, you know, has his individual appointments, and then earlier we were having family sessions every week pretty much I think for a while, and then back down to every two weeks. And now it's only like once every 4–6 weeks, I think like every month or a little less than that. So yeah, because it was, you know, him navigating his personal recovery journey, and then us navigating the family situation, you know, in addition to that*. - Alana, Chris's mother
*I was getting the money incentive when I was part of {treatment program], so she talked to [my caregiver] and we decided that when I get my shot, she'll send me $20. so she does that, and I also just talk to her on a regular basis and let her know what's going on*- Helen (age 25–30)	*I do, but it's an hour away and that's an issue for me or else I would have been down there before now. It's not that I can't go but I don't like driving that far really often. I will probably go now and see her since you brought this up*. - Christine, Helen's grandmother
*She's been in contact with my counselor, they talk. She gives me updates. My insurance company cut my time short and I called her and she hopped on the phone with my insurance company to try to get something done. She makes sure that I'm on my shit*- Lewis (age 31–36)	*She felt kind of defeated, and didn't want to go and I talked to her, I said as much as I would like for you to come home. I said, I would like you to stay in more, because it's gonna benefit you. And I know you miss your babies. But if you go ahead and sacrifice and commit yourself, then you will never have to leave your babies again*. - Jackie, Lewis's partner

### Theme 4: YAs can identify CSOs who are supportive and encouraging of treatment and treatment goals even in the face of CSO barriers and challenges

All 10 YAs reported being in alignment with their CSOs on treatment goals. Treatment goals most commonly included self-sufficiency and long-term recovery (*n* = 5, e.g., Helen and Christine). Other goals related to childcare and custody (*n* = 1), medication as treatment for OUD (*n* = 1), and housing stability (*n* = 1) (Table 7).

**Table 7:** Exemplars for Theme 4: YAs can identify CSOs who are supportive of their treatment goals.

**YA**	**CSO**
*But the way she is toward me is like she's the one person who I can say would never give up … she's the one person where I could say if I ever need anything, I can call grandma because I know she's got my back...And when I got to when I started using everything, she was the most supportive of helping me get back on the right path because she was always there … so that's like our relationship is like rock solid*. - Seth (age 25–30)	*Not that the other ones weren't involved and caring but this particular time, seems like he has more people in his corner and they want to see him do right and get on that path that he needs to get on*. - Barbara, Seth's grandmother
*I think my grandma's main goal is just to see me stay clean and get things accomplished for my life...I wanna stay clean and actually develop a life....We have a really good relationship. my whole life she's been more my mother than my grandma, and she idk she loves me. I don't think I can do any wrong in her eyes and she's very very proud that I'm clean and even when I relapsed she just wants to see me do better*- Helen (age 25–30)	*I feel good where she is now. I'm glad she's clean and sober. I hope she stays that way and I think if she had a job and a place to live she probably would...I've never been asked to go to a family... I didn't know they had that. She's never asked me to go to anything but I would if she did, you know. I would do anything to help her*. - Christine, Helen's grandmother

Despite all CSOs being supportive of treatment, seven mentioned significant challenges or barriers to being involved with their YA's treatment. Barriers included logistical constraints such as having a too-busy daily schedule (*n* = 4; e.g., Jackie), the treatment center being far away (*n* = 1; e.g., Christine), and their own physical health issues that made traveling for visits difficult (*n* = 1; e.g., Karen). CSOs also mentioned emotional challenges such as feeling burnt out in their caregiving role (*n* = 3; e.g., Jackie), believing that their YA was rejecting the treatment being offered (*n* = 1), and feeling disappointed and angry in their loved one (*n* = 1). One CSO mentioned their own lack of knowledge about addiction and the treatment system as a barrier to their involvement (Table 8).

**Table 8:** Exemplars for Theme 4: CSOs experienced barriers to participating in services.

**CSO**
*I do [wish I knew more about her treatment], but it's an hour away and that's an issue for me or else I would have been down there before now. It's not that I can't go but I don't like driving that far really often. I will probably go now and see her since you brought this up*. - Christine, Seth's grandmother
*My biggest challenge sometimes being the fact that I take care of so many people, we live off my husband's income. And I try to do some jobs on the side. And with me being, when I - just recently with me being sick, I'm not able to go to work right now. So, it cuts into our money, but I'm still trying to give [YA] what I can. And sometimes I don't think she realizes that, and she kind of gets upset. So, I'm trying when I can. And then sometimes [YA] I don't think realizes certain things. She just kind of expects us to continue taking care of her, and I need her to realize there is life out here. Just ‘cause you're in there, life still is going on outside of rehab. Things happen out here*. - Jackie, Lewis's partner
*Yeah, it's a lot. It's overwhelming at times. But they‘re my grandbabies and I love them and I would prefer them to be with me than in foster care. So I'll do what I have to do. And I do a lot of praying. Keep me in good health so I can keep the strength that I need to do it. Sorry, I'm getting emotional...It's taking a toll on the whole family. Not just us. It really disrupts the whole family*. - Karen, Ann's mother

### Theme 5: YAs hold accurate perceptions of their CSOs' MOUD attitudes and beliefs

YAs understood their CSOs' feelings and opinions about MOUD treatment, which were either positive or mixed though generally positive. We previously reported on MOUD goal alignment as perceived by YAs themselves, finding that most YAs believed their CSO felt similarly about MOUD as they did (23). Here we provide evidence that YAs perceptions were accurate, even when CSOs held more complex mixed views about MOUD. Nine YAs accurately described their CSO's attitudes toward MOUD; one CSO was not asked about this. For four dyads, CSOs confirmed positive opinions about MOUD. The most cited positive benefit of MOUD by both YAs and CSOs was relieving cravings and withdrawal symptoms to facilitate reducing and stopping opioid use. The remaining five CSOs confirmed mixed feelings about MOUD. These CSOs recognized the positive benefits of MOUD in general and for their YA specifically while holding concerns and worries. YAs were able to identify CSO concerns about MOUD, which were often rooted in unknowns about long-term use, addictive properties of MOUD, and importance to recovery (Table 9).

**Table 9:** Exemplars for Theme 5: YAs hold accurate perceptions of their CSO's MOUD attitudes and beliefs.

**YA**	**CSO**
**Positive attitudes toward MOUD**	
*I guess she like, she loves it because it helped me and hasn't had a negative side negative side effects on me…once it was explained to her it made a lot more sense*. - Grayson (age 25-30)	*I think that this treatment, the Subutex is offered right away…You shouldn't be waiting, going to rehab after rehab, it should be the first option. The Subutex, I mean. Because I really think that's key. Because they're not in constant withdrawal, or having to take constant shots of methadone, or stuff like that. It's once a month, out of mind, out of sight, and then you go on through life*. - Lucy, Grayson's mother
*Yeah man, she said, “You know if that's what you need to do to get some clean time, then that's what you need to do. As long as you're taking it as prescribed, I still see you as clean.”*- Kelly (age 25–30)	*This is the first time in treatment that she's been on Suboxone, so I'm hoping that that will help her to be able to stay clean because she's never been on something to help her stay clean. I've, I mean every person is different, so I worry that then she would have to come off of suboxone 'cause I know suboxone can be in an addicting form too as well. So, I've talked with her about that, but she is really, she's been addicted for so long she has a really hard time staying clean, and a lot of it is, I feel a lot of her stuff is emotional in staying clean. She gets in a fight with her mom, she tends to go back to using really quick, so I know the suboxone can help in that way in her staying clean. So, think yeah, that's a good thing for her*. - Janis, Kelly's mother
**Mixed attitudes toward MOUD**	
*So, the medication part was more of a, you know, if you feel like you can get off the medication, she would say okay, you know what I mean. Like she's not a hundred percent with taking so many medications, but if I need it need it, then that's something different, you know what I mean?*- Seth (age 25-30)	*Well really, you know, I know that he's on a couple of medications and, you know, I guess I kind of have the question of is that, you know, a permanent thing? Is he gonna need to be on medications, you know, indefinitely? Is there an end point for that? And that's kind of just a minor concern, but we haven't talked a whole lot about that as far as what the long-term expectation is with that. But I guess because there's, you know, there's a part of me I guess that, you know, I want to see him free of the need for any substances, so you know, even like regular prescription medications if, you know, if he could live without those. But I definitely want him to have what he needs to be able to live, you know, the drug free lifestyle, so..*. - Barbara, Seth's grandmother
*Yeah, she's 50/50 with it. And that's one of the reasons why I'm 50/50 with it too, for real….She just wants me to be careful when making a decision and doing it. She basically the same way, she wanna know more about the shot before I actually do it, you know what I'm saying?*- Owen (age 25–30)	*It sounds okay. I mean if it's going to help him, then I'm all for it. But it sounds like it's okay, but him just being home for the past month, I don't know… this is something I probably have to talk to him about like again- how do you actually feel? Do you feel like you're gonna need that shot maybe once a month, you know, 'cause he's not on nothing now. And I'm just wondering is it really necessary 'cause like you said I'm not really confident about answering that question, so I'm not really sure. But if it works for him, if it's something he wants to do, then that's okay I guess, but the real question is, is he going to really need it?*- Caitlin, Owen's partner

## Discussion

Through identifying qualitative themes, we learned lessons that can be leveraged to improve rates of CSO involvement in OUD treatment for YAs. In this sample, YAs identified CSOs who were encouraging and supportive of their treatment and treatment goals, with relationships described by both YAs and CSOs as connected and motivating. CSOs valued participating in services along the treatment services continuum from facilitating treatment entry to participating in systemic family therapy sessions. Moreover, CSOs identified family involvement in services as essential and experienced personally meaningful benefits from participating. Nonetheless, even in this sample of motivated and engaged CSOs, barriers to participating in services for their loved one persisted, consistent with barriers experienced by CSOs documented in the literature (29, 32).

It is encouraging that when providers collaborated with patients to identify CSOs to participate in treatment, and those CSOs were engaged in services, patients and CSOs alike identified positive individual and relational outcomes. This is not surprising given the vast literature on the effectiveness of family therapy and family involved treatments for substance use disorders (14, 40). YAs reported feeling more motivated for treatment and CSOs described both feeling relieved to be informed of their loved one's treatment and invested in learning about addiction and stigma to better support them. Moreover, greater connection and more open and nonjudgemental communication were identified by YAs and CSOs alike as an outcome of CSO supporting and participating in services.

Yet, our findings extend our previous research indicating there is ample room to grow in leveraging relationships to support OUD treatment engagement and retention for YAs. We previously reported on findings suggesting YAs value family involvement in services and preferred greater CSO involvement in their own OUD treatment (23). Results of the current study indicate CSOs hold similar beliefs. CSOs described family responsibility to support loved ones in treatment, yet very few were targeted by providers for interventions like family therapy or family recovery planning. This is an unfortunate trend (24) reflecting ongoing barriers to family involvement (25, 29).

The current findings may reflect provider reluctance to engage family members in services for YAs. Previous research describes hesitations among providers to collaborate with family members for YAs due to misinformed assumptions about developmental needs [e.g., (25, 31)]. Despite recruiting from treatment programs that value family involvement in services, only one dyad reported family members participating in family therapy sessions despite all 10 CSOs being invested to support their loved one. In order to increase CSO involvement in services, providers must see the benefit to CSO collaboration and possess the knowledge, skills, and confidence to use family therapy interventions.

These findings advocate for routine CSO involvement in services and recovery planning, and highlight the ongoing need for provider, patient, and family-facing resources to increase the availability and accessibility of evidence-based family interventions (25). Efforts to disseminate existing evidence-based pragmatic provider training resources designed to enhance competence and confidence in collaborating with CSOs and inviting their participation in treatment must be prioritized (e.g., https://drugfree.org/clinical-training-academy/). Furthermore, CSOs in this sample suggested the treatment services were difficult to access for a number of personal and logistical reasons. Better disseminating family-facing supports designed to provide education on navigating the treatment system and advocating for family involved care is essential (e.g., https://drugfree.org/article/navigating-the-treatment-system/).

### Study strengths and limitations

The strength of this qualitative study lies foremost in the rigorous data collection and analysis procedures. With the vulnerabilities of the participant population and the sensitive nature of the interview topics in mind, interviewers were afforded flexibility to build rapport while the structure of the interview guide ensured all important topics were covered. Data collection was followed by innovative analysis procedures that blended traditional thematic content analysis with matrix analysis, allowing for analysis of key themes and the frequency with which themes and subthemes emerged across family members and dyads.

A main study limitation is possible self-selection bias influencing emerging themes. More than half of the CSOs who were contacted to participate in the current study were unreachable or declined to participate. Is it possible, if not likely, that CSOs who agreed to participate in the study were fundamentally different with respect to their level of involvement in their Yas' treatment and beliefs about the importance of family involvement in treatment.

### Future directions

Future directions include developing and disseminating (1) training resources designed to enhance provider knowledge, skill, and confidence collaborating with family members and inviting their participation in treatment; and (2) family-facing resources on navigating the substance use disorder treatment system. Future research should also prioritize learning more from YA OUD providers about their attitudes, beliefs, and experiences offering services to CSOs.

## Data Availability

The original contributions presented in the study are included in the article/Supplementary material, further inquiries can be directed to the corresponding author.
