# Supplementary material for: Lessons learned from concerned significant others: a qualitative analysis on involvement in services for young adult opioid use disorder
Source: Front Public Health. 2025 Jul 17;13:1512529. doi: 10.3389/fpubh.2025.1512529 (PMC12310619; doi:10.3389/fpubh.2025.1512529)
Supplement: Supplementary file 1 [file Data_Sheet_1.docx]

Appendix A

**CSO Qualitative Interview Guide**

**Intro Script**

Thank you for taking some time to speak with me today. As you learned during the interview consent process, our research team is hoping to better understand how young adults can be supported during treatment and recovery for opioid use. We’re especially interested in the role that relationships might play in treatment, and how opportunities for positive family involvement might benefit young adults during treatment and recovery. This interview is not intended to influence you or your loved one’s treatment decisions in any way, nor are we hoping to change any views you might hold regarding your involvement in treatment. We hope only to better understand your perspective on these topics, which will help us learn how we might improve the quality of care for young adults.

With your permission to continue, this interview will last between 45 minutes and 1 hour. You will be asked some questions about your loved one’s treatment and recovery planning, particularly when it comes to family involvement. If there are questions you are uncomfortable answering, you can always decline to respond. Even though you signed a consent to allow us to interview you, that does not mean you have to do the interview—you can refuse right now, or stop the interview at any time during the interview. And please note that anything you share with me today will be kept entirely confidential, including from your loved one and their treatment provider. Your identity will also be removed from your responses before they are later reviewed by our research team as part of our current study.

I want to quickly note that while the idea of “family” means something different to everyone, today we’ll be using the term “family” to refer to the individuals, or network of individuals, who are most central to your loved one’s life. While this may include family of origin such as parents, grandparents, or siblings, family may also consist of other close individuals such as a partner or trusted friend.

Do you have any questions about our research or the interview we’ll be doing today?

**Intro to OUD and Relationship with Young Adult**

As I mentioned earlier, our interview will mainly focus on family involvement in treatment and recovery for Opioid Use Disorder, or OUD. That term, OUD, is it something you’ve heard before?

Opioid Use Disorder, or OUD, is defined as the chronic use of opioids that causes someone significant distress or impairment. OUD often interferes with one’s ability to fulfill responsibilities at work, school, or home.

Now, I want you to think about your relationship with [loved one name]. This is your loved one, who as you know is struggling with Opioid Use Disorder. To get us started, I was hoping you could share some details about your relationship with them.

- *Follow up on any relevant details*
- On a scale of 1-10, how close is your relationship with your loved one?
- How much time do you spend together each week?
- What are some activities you enjoy doing together?
- Has your relationship with your loved one changed since the start of treatment?

**Family History**

- Is this the first time your loved one has been in treatment for substance use?
  - (If no) When else has your loved one been in treatment for substance use?
    - Were you involved at all in their previous treatment episodes?
    - Do you think that those treatment experiences were successful? Why or why not?
- Do you know anyone else who’s been in treatment for substance use?
  - *(If self)* Can you share some more about your experiences in treatment?
  - *(If others)* Can you share what it was like to see them in treatment for substance use?

**Current Treatment Plan**

- When did your loved one start their current course of treatment?
- How are you feeling about their current treatment?
  - Are there parts of the treatment you feel confident in?
  - Are there parts of the treatment you feel nervous about?
  - (If not yet mentioned) How do you feel about medication being a part of treatment?
    - (If applicable) How do you feel specifically about buprenorphine (Suboxone, Subutex), methadone, and extended release naltrexone (Vivitrol)?
- Have you and your loved one had any conversations regarding their treatment?
  - (If yes) How did those conversations go?
  - How do you think your loved one is feeling about their treatment plan?
  - Does anything stand out about the conversations you and your loved one have shared about their treatment?
  - *(If no)* What do you think has prevented you two from having those conversations?
    - Do you think it would be helpful to have a conversation with your loved one about their treatment? Why or why not?
- How did your loved one decide to pursue their current treatment plan?
  - Were you included in your loved one’s decision-making process?
    - Was there anyone else your loved one included in their decision-making process?
  - Are there any other factors that you think shaped your loved one’s treatment decisions?

**Treatment Goals**

Next, I want to talk a little bit about goals for treatment and recovery.

- Do you have any goals for your loved one in treatment? (Or in other words, do you have a preferred outcome in mind for your loved one as a result of their treatment?)
  - (If so) What are those goals?
  - Do you feel like your loved one is on track to meet those goals? Why or why not?
  - (If necessary) What is your definition of a ‘successful recovery’ for your loved one?
- Do you think you and your loved one have similar goals for them in treatment?
  - (If yes) In what areas do you think your goals for treatment are aligned?
  - (If no) How do your treatment goals for your loved one differ from the treatment goals they have for themselves?
- How about goals outside of treatment, do you think you and your loved one are aligned there as well?
  - Where do your goals for your loved one align with the goals they have for themselves? Where do they differ?

**Involvement in Treatment**

I was hoping we could shift our discussion toward involvement in treatment, and what that looks like or could like. To start, are you currently involved in your loved one’s treatment?

*If involved:*

- Going into detail, can you describe to me what your involvement in your loved one’s treatment looks like?
  - What has been positive for you about being involved in treatment?
  - What has been challenging for you about being involved in treatment?
  - Is there anything you wish were different regarding your involvement in treatment?
- What were your reasons for becoming involved in treatment?
- How do you think your loved one feels about you being involved in treatment?
  - How do you think they benefit from you being involved in treatment?
  - Do you think your involvement in treatment could be challenging for them in any way?
- Have you had any conversations with your loved one where you directly discussed your role/involvement in their treatment?
  - (If yes) Did talking to your loved one change how you think about your role in their treatment? (Or in other words, do you feel like you better understand your role as a result of those conversations?)
  - (If no) Do you think it could be helpful to have a conversation like that with your loved one? Why or why not?

*If not involved:*

- Has it been hard to not be involved in your loved one’s treatment??
  - In your view, what are the pros of not being involved?
  - In your view, what are the cons of not being involved?
  - How do you think your loved one feels about you not being involved in treatment?
- Do you see getting involved in treatment in the future as a possibility?
  - If that’s something you are interested in, what barriers currently stand in the way of you getting involved?
  - What would have to be different in order for you to be interested in being involved? To be able to be involved?
- How would it feel to be a part of your loved one’s treatment?
  - How do you think your loved one would feel about you getting involved in treatment?
  - Have you discussed the possibility of getting involved with your loved one?

*Whether involved or not:*

- Time is of course a major factor when it comes to being involved in treatment… can you give me a sense of the demands on your time these days, what your schedule is like?
  - How do you think the demands on your time factor into your ability to be involved in treatment?
- Is it important for you to be a source of support for your loved one during this time? Why or why not?
  - *(If yes)* Outside of being involved in treatment directly, how do you support your loved one during this time?
    - (If necessary) What do you think makes your loved one feel most supported by you?
- Is there anyone else that you know has been involved in your loved one’s treatment?
  - What is their role in treatment?
    - *(If applicable)* In what ways is their role different than your own?
    - How do you think your loved one feels about their involvement in treatment?
    - How do you feel about their involvement in treatment?
  - Is there anyone not involved in your loved one’s treatment that you think should be?
- Beyond your loved one’s current treatment plan, do you see yourself being involved in their long-term recovery?
  - What do you think that involvement might look like?
  - Have you discussed the prospect of being involved in the future with your loved one?
    - (If yes) How did those conversations occur? (As part of treatment or informally outside of treatment?)

**Attitudes towards Family Involvement**

As we’re approaching the end of our time, I was hoping we could discuss your thoughts on the role of family in substance use treatment and recovery.

- Do you think family involvement is important for helping young people in their recovery from substance use? Why or why not?
  - What challenges do you think might be faced by those attempting to recover without family support?
  - In a perfect world, what do you think the role of family in treatment should look like?
    - (if client asks, clarify that this can be for their family specifically or in general)
- Some people believe that since patients are the ones with OUD, they should be the ones responsible for their treatment – that nobody else, including family, is responsible for helping them with treatment. What do you think about that (the idea that family and others have no obligation to help with treatment)?
- Have your thoughts on family involvement in treatment and/or recovery changed since the start of treatment?
